# The ‘lost generation’ in adult psychiatry: psychiatric, neurodevelopmental and sociodemographic characteristics of psychiatric patients with autism unrecognised in childhood

**DOI:** 10.1192/bjo.2023.13

**Published:** 2023-05-24

**Authors:** Johan Nyrenius, Jonas Eberhard, Mohammad Ghaziuddin, Christopher Gillberg, Eva Billstedt

**Affiliations:** Gillberg Neuropsychiatry Centre, Institute of Neuroscience and Physiology, Sahlgrenska Academy, University of Gothenburg, Sweden; Adult Psychiatry Clinic Helsingborg, Region Skåne, Sweden; and Department of Clinical Sciences Lund/Clinical Sciences Helsingborg, Lund University, Sweden; Adult Psychiatry Clinic Helsingborg, Region Skåne, Sweden; and Department of Clinical Sciences Lund/Clinical Sciences Helsingborg, Lund University, Sweden; Department of Psychiatry, University of Michigan, Michigan, USA; Gillberg Neuropsychiatry Centre, Institute of Neuroscience and Physiology, Sahlgrenska Academy, University of Gothenburg, Sweden

**Keywords:** Autism spectrum disorder, adults, psychiatry, comorbidity, neurodevelopmental disorders

## Abstract

**Background:**

Patients with ‘underlying’ autism spectrum disorder (ASD) constitute a significant minority in adult out-patient psychiatry. Diagnoses of previously unrecognised ASD are increasing in adults. Characteristics of patients with autism within adult out-patient psychiatry have not been sufficiently explored, and there have not been any systematic comparisons of characteristics between patients with and those without autism within adult out-patient psychiatric populations.

**Aims:**

To examine psychiatrically relevant characteristics in autistic adult psychiatric out-patients, and to compare the characteristics with non-autistic adult psychiatric out-patients.

**Method:**

We assessed 90 patients who were referred to a Swedish psychiatric out-patient clinic and screened for ASD during 2019–2020. Sixty-three patients met the DSM-5 criteria for ASD or ‘subthreshold’ ASD. The 27 who did not meet the criteria for ASD were used as a comparison group. Assessments were made with structured and well-validated instruments, including parent ratings of developmental history.

**Results:**

No differences were found between the groups regarding self-reported sociodemographic variables. The ASD group showed a higher number of co-occurring psychiatric disorders than the non-ASD group (*t*_(88)_ = 5.17, 95% CI 1.29–2.91, *d* = 1.19). Functional level was lower in the ASD group (*t*_(88)_ = −2.66, 95% CI −9.46 to −1.27, *d* = −0.73), and was predicted by the number of co-occurring psychiatric disorders.

**Conclusions:**

The results underscore the need for thorough assessment of psychiatric disorders in autistic patients in adult psychiatric services. ASD should be considered as a possible ‘underlying’ condition in adult psychiatry, and there is no easy way of ruling out ASD in this population.

Autism, or autism spectrum disorder (ASD), is a developmental disorder with onset in early childhood, characterised by marked social communication deficits with repetitive behaviours, restricted interests and sensory abnormalities.^1^ ASD affects about 1–1.5% of children.^2^ Several studies have shown that ASD persists into adult age^3,4^ and can be regarded as a lifelong condition, with prevalence rates in adults comparable to those in children.^5,6^ Recent studies have also suggested that ASD is relatively prevalent in adult psychiatric out-patient settings. Prevalence has been estimated as 16% in out-patients with depression,^7^ 15–27% in out-patients with eating disorders^8^ and substantially differing estimates (ranging from 1.4%^9^ up to possibly 19–20%^10,11^) in general psychiatric out-patient services. However, prevalence estimates in adult psychiatric populations are methodologically difficult, as all instruments available for screening and diagnostics of ASD suffer from poor psychometric properties in adult psychiatric populations.^12^ The low specificity in screening and diagnostic instruments could cause an overestimation of autism in prevalence studies.^13^

## Delayed diagnosis

A recent systematic review^14^ reported that, between 2012 and 2019, the global mean age at ASD diagnosis was 60.48 months (range 2.5–19.5 years). Commonly reported factors that might contribute to older age at diagnosis include the occurrence of additional diagnoses such as attention-deficit hyperactivity disorder (ADHD), dyslexia or Tourette's syndrome;^15,16^ presence of complex verbal ability/higher verbal IQ;^17^ occurrence of adverse life experiences;^18^ being female (females in some studies tend to be older at time of diagnosis)^19^ and the type of ASD (without co-occurrence of intellectual disability or Asperger-type symptoms).^20^ Diagnosis of ASD in adulthood is an important clinical issue. Some researchers have used the term ‘lost generation’ to describe the group of autistic adults who missed receiving adequate support in their early years because they were never accurately diagnosed in childhood.^21^ Various experiences have been reported by persons receiving an ASD diagnosis in adult age, ranging from mostly positive to occasionally negative,^22^ positive in general but painful to adjust to its consequences,^23^ or – rarely – all negative.^24^ Examples of positive reactions are relief that their feelings of being different were validated.^22^ Anger and shock are negative reactions that have been associated with delayed diagnosis.^22^ Although most studies report inadequate availability of supportive services for autistic adults,^22^ relatively little is known about any clinical effects on well-being or psychiatric symptoms from receiving an ASD diagnosis.^22^

## Co-occurring conditions

Foremost among the factors that contribute to the initial diagnosis of ASD in adulthood is the presence of a co-occurring condition, which affects outcomes to a greater extent than the ASD itself.^24^ Clinically impairing ASD is almost always associated with other neurodevelopmental and psychiatric disorders; co-occurring conditions are the rule rather than the exception.^25^ The most prevalent co-occurring psychiatric disorders are depression, anxiety disorder and bipolar disorders.^26–28^ There are conflicting opinions about the co-occurrence of ASD and psychosis, in part because distinguishing psychotic features from features of ASD is very difficult.^29^ The rates of psychotic symptoms in ASD range from 2 to 10%.^26,30,31^Autistic adults suffer from psychiatric disorders to a greater extent than non-autistic adults, at least up to middle age.^27,28^ Two recent meta-analyses on co-occurring mental health disorders (including ADHD) reported higher rates in autistic adults than in the general population,^28,32^ which could be a contributing factor to the high prevalence of ASD in adult psychiatric settings.^11^

Of other neurodevelopmental disorders co-occurring with ASD, the most common is ADHD.^21,28,31^ Tic disorders are also very common (22–50%), as are motor difficulties and speech and language difficulties.^31,33,34^

Most studies on co-occurring conditions have focused on the general ASD population; studies on autistic adults within out-patient psychiatric settings are considerably scarce and limited to only a few published articles.^27,30,35,36^ To our knowledge, no study has so far compared patients with and without ASD within an adult psychiatric out-patient population. Such a comparison would possibly highlight differences in psychiatric profiles and care needs between patients with and those without ASD in adult psychiatry. More knowledge is needed about autistic persons who are of normal intelligence, who are undiagnosed until adulthood. This ‘lost generation’^21^ seems to constitute a significant minority of the adult out-patient psychiatric population.^10,11^

## Aims

The primary aim of the study is to examine psychiatric and neurodevelopmental disorders, sociodemographic characteristics, history of alcohol/drug consumption, functional level, ASD symptoms and developmental history in a sample of autistic patients, screened from a clinical adult psychiatric out-patient population.

The secondary aim is to compare the factors listed above between the sample of autistic patients and a sample of non-autistic patients from the same clinical adult psychiatric out-patient population.

## Method

### Procedure

New patients attending a specialist psychiatric out-patient clinic within the public healthcare system in Helsingborg, Sweden, were screened for ASD between 1 January 2019 and 31 December 2020. Patients are referred to the clinic via primary healthcare settings or by self-referrals. Self-referrals are assessed by experienced specialist nurses, and a team decision of accepting or not accepting self-referred patients is made by a nurse and psychiatrist. The large majority of psychiatric patients in Sweden are treated within the public healthcare system. In Helsingborg, Sweden, responsibility is divided between primary healthcare and specialist psychiatry. Specialist adult psychiatry is responsible for out- and in-patient treatment of moderate-to-severe psychiatric disorders, defined as as severe crisis reactions, eating disorders, moderate-to-severe or treatment-resistant depression, bipolar disorders, moderate-to-severe anxiety disorder, substance use disorders, severe personality disorders and psychotic disorders. It is also responsible for assessment and treatment of ADHD, and assessments of ASD. Patients with less severe psychiatric disorders are treated in primary healthcare settings, and the rules regarding which patients get access to specialist psychiatry services are strictly regulated.

Screening was performed at the psychiatric assessment unit, where all new catchment area patients who have not had any psychiatric contact during the past 6 months are assessed, except for those referred for clear psychosis, substance use disorders and those aged ≥67 years. New patients at the substance use disorders unit were also screened according to the study protocol, but the psychosis units (comprising 1% of all new patients during 2019–2020) and the geriatric (age ≥67 years) psychiatry unit (comprising 7% of all new patients during 2019–2020) were excluded. The catchment area consisted of approximately 200 000 people aged 18–66 years. Out of 1030 new patients during 2019 and 2020, a screened sample of 90 patients consented to participate in the study, hereafter called the ‘participants’. Forty-eight participants who had positive ASD screening results were drawn from systematic screening conducted during November 2019 to October 2020, comprising 25% of the total number who had positive ASD screening results during this period. A further 33 participants who had positive ASD screening results were drawn from unsystematic screening conducted during January to October 2019. Nine participants who had negative ASD screening results were drawn from the systematic screening. The screening period was shortened because of the COVID-19 pandemic and the screening process has been described previously.^11^ All participants (*N* = 90) were subject to in-depth assessments with the instruments listed below.

### Ethics approval and participant consent

The authors assert that all procedures contributing to this work comply with the ethical standards of the relevant national and institutional committees on human experimentation and with the Helsinki Declaration of 1975, as revised in 2008. All procedures involving human patients were approved by the Regional Ethics Review Board in Lund, Sweden (reference number 2018/740). All participants provided written informed consent.

### Participants

The total sample of 90 participants (36 males, 54 females; age range 18–65 years; age mean 31.0 years, s.d. = 10.5; IQ estimate mean 99.9, s.d. = 11.7) were first divided into three groups: ASD, subthreshold ASD and non-ASD. The ASD group (*n* = 52) met the full DSM-5 criteria for ASD (20 males, 32 females; age mean 30.6 years, s.d. = 9.8). The subthreshold ASD group (*n* = 11) met two rather than all three of the A-criteria and at least two of the B-criteria (seven males, four females; age mean 38.6 years, s.d. = 11.7). The non-ASD group (*n* = 27) did not meet the criteria for ASD or subthreshold ASD (nine males, 18 females; age mean 29.0 years, s.d. = 10.4). In terms of criteria for Asperger syndrome, 22 of the 52 participants in the ASD group (and none in the other two groups) met Gillberg & Gillberg's criteria for Asperger syndrome;^37^ 31 of the participants in the ASD group and seven of the 11 participants in the subthreshold ASD group met the criteria for Asperger syndrome from the ICD-10.^38^ Two participants (both having positive ASD screening results) had received an ASD diagnosis before the study entry: one female who had received her ASD diagnosis in her late teens and one male who had received his ASD diagnosis in his early 40 s. Both met the full DSM-5 criteria in the study and were included in the ASD group. The non-ASD group was composed of both participants who had positive ASD screening results (*n* = 19) and those who had negative ASD screening results (*n* = 8; one participant that had a negative ASD screening result received a research diagnosis of ASD). According to the screening results, 65% of the screening responders had positive ASD screening results and 35% had negative ASD screening results;^11^ the non-ASD group could be considered representative of patients without ASD from the study population. Specific data on race/ethnicity were not recorded.

### Measures

#### Screening instruments

The initial screening instrument was the Ritvo Autism Asperger Diagnostic Scale – Revised^39^ (RAADS-R), an 80-item (total score ranging from 0 to 240) self-rating scale, designed as a screening tool for ASD in adults aged ≥18 years. The scale has shown fair to good psychometric properties. To limit the number of false negatives, the cut-off was set to 50, which is lower than what is recommended for clinical use. A cut-off of 50 points has been shown to give a sensitivity of 96% and a specificity of 82% in a non-psychiatric sample.^40^ Because of low response rates, the screening instrument was changed on 1 November 2019, to the shorter RAADS-14 Screen,^41^ a 14-item (total score ranging from 0 to 42) self-rating scale derived from the RAADS-R. We used the recommended clinical cut-off for RAADS-14 (14 points), which has been shown to give a sensitivity of 97% and a specificity of 64% in psychiatric populations.^12,41^ A RAADS-14 score of ≤6 was originally intended as a criterion for inclusion in a comparison group.

#### Assessment of psychiatric conditions and other neurodevelopmental conditions

The Mini-International Neuropsychiatric Interview (MINI) version 7.0.1 with ADHD supplement^42^ is a short, structured diagnostic interview. The interview is designed to identify occurrence of the most common psychiatric disorders according to the DSM-5. The sections on suicidality and substance/alcohol use disorders were replaced by a questionnaire about suicidality, the Alcohol Use Disorders Identification Test^43,44^ (AUDIT) and the Drug Use Disorders Identification Test^45^ (DUDIT). Tic disorder was diagnosed in participants showing clear tics during the interview and/or self-reporting tics according to DSM-5 criteria.^1^

#### Assessment of functional level

The Global Assessment of Functioning^46^ (GAF) is an internationally widely used assessment of psychosocial functioning level ranging from 0 to 100, with a higher score suggesting better psychosocial functioning.

#### Assessment of sociodemographic/socioeconomic characteristics

A structured self-rating questionnaire was completed by the participants, covering living conditions, relationships, educational background, vocational status, economy, contacts with social services, healthcare contacts including psychiatry, prior assessments and medicine use.

#### Assessments of alcohol and narcotics consumption

The AUDIT^43^ is a short self-rating scale for identification of hazardous or harmful alcohol use. The scale consists of ten items and scores range from 0 to 40. The cut-off has been set as ≥8 for males and ≥6 for females.^44^

DUDIT^45^ is a short self-rating scale for identifying persons with drug-related problems. The scale consists of 11 items and scores range from 0 to 44. Scores indicating drug-related problems for men are ≥6 in ages ≥26 years, and ≥7 in ages ≤25 years. For women, drug-related problems are indicated at a score of ≥2 for ages ≥26 years, and ≥3 for ages ≤25 years.

#### Assessment of cognitive functioning

Three subtests (Matrix Reasoning, Coding and Symbol Search) from the Wechsler Adult Intelligence Test, Fourth Edition (WAIS-IV)^47^ were administered. The subtests Matrix Reasoning and Coding have been shown to accurately estimate IQ level.^48^ Results from the subtests Coding and Symbol Search were used to calculate the processing speed index (PSI) score.

#### Parent questionnaire

The Five-to-Fifteen – Revised^49^ (FTF) questionnaire was given to the participants for forwarding to any one of their parents, unless the participant (for any reason) was unwilling to involve their parents. The parents were instructed to answer the questionnaire based on how the participants presented at 17 years of age. The FTF measures difficulties in children and youth aged 5–17 years. One study has shown the FTF to be useful when administered retrospectively to relatives of adults;^50^ another study has shown that 24 out of the original 181 items show good agreement over time, and therefore should be selected for use in adults.^51^

#### Diagnostic assessment of autism

The Asperger Syndrome (and high-functioning autism) Diagnostic Interview^52^ (ASDI) is a semi-structured interview consisting of 20 questions, with scores ranging from 0 to 40. The interview is designed to be used by a clinician as an aid in preliminary diagnostic decisions. It has shown good inter- and intrarater reliability and acceptable validity.^52^ The ASDI was not used to diagnose ASD in this study, but was used as a structured means of gathering information about symptoms of ASD. As previously stated, the various instruments available for diagnosing ASD show inferior psychometric properties when applied to adult psychiatric patients.^12^ Instruments considered as the ‘gold standard’ in children and adolescents do not show the same precision in adult psychiatric populations.

All available data from the in-depth assessments, including those obtained with the ASDI, FTF and other instruments, and clinical assessments of the participants, were used to assess ASD diagnosis (research diagnosis) according to DSM-5 criteria. All assessments, and all decisions on presence (or not) of ASD, were performed by clinicians with extensive clinical experience diagnosing ASD. Conjoint validation of the research diagnoses was performed by four of the authors (J.N., M.G., C.G. and E.B.), in a subsample of five participants. Details regarding the ASD assessments (including conjoint validation) have been published previously.^11^

#### Other instruments

The results from the screening questionnaires have been presented in a previous study.^11^ Questionnaires about suicidality; non-suicidal self-injury (Functional Assessment of Self-Mutilation^53^); avoidant/restrictive food intake disorder symptoms (Nine-Item Avoidant/Restrictive Food Intake Disorder Screen^54^) and paraphilias, sexual orientation and gender dysphoria were also administered, but results will not be presented here.

### Data analysis

#### Sociodemographic characteristics

Vocational status, educational background, social relationships, living situation, current and past support from social services and history of contacts with psychiatric services were chosen for analysis, as they are (according to clinical practice) commonly associated with psychiatric disorders.

#### Autism symptoms and parent-rated developmental history

Diagnostic tools and instruments for assessing autism suffer from poor psychometric properties in adult psychiatric populations.^12^ Presenting the distribution of clinically assessed ASD symptoms in the two groups and analysing any between-group differences is therefore of interest, particularly in understanding how common autism symptoms are in participants without ASD. It is also of interest to study any between-group similarities and differences in parent-rated developmental history, as neurodevelopmental disorders have been proposed to be very common in adult psychiatric populations.^10^

#### Psychiatric disorders

Variables used when analysing psychiatric disorders were current diagnoses, according to the MINI, in the different DSM-5 diagnostic categories. These variables were chosen because more detailed information about psychiatric diagnoses would not be meaningful because of diagnostic overlap. Number of current psychiatric non-mood disorders was used as a measure of degree of co-occurring psychiatric disorders; mood disorders were left out as they are very common in all psychiatric populations.

#### Outcome

GAF score was chosen as outcome measure because it is supposed to measure degree of disability at the time of participation. Analysis of GAF score was made with common factors associated with functional level, such as age, cognitive measures (processing speed and IQ), presence of ADHD, number of current psychiatric non-mood disorders and degree of ASD (expressed as ASDI score).

### Statistical analyses

All statistical analyses were made with IBM SPSS for Windows, version 25. The statistical significance criterion was set *a priori* at (*α*-level) *P* = 0.05. Between-group comparisons were performed with *t*-tests or one-way analyses of variance for normally distributed variables. For categorical data, *χ*^2^-tests or Fisher's exact test (for 2 × 2 tables) were used. Regression analysis was performed for estimating relationships between the outcome variable GAF score and different predictors. Correlations with GAF score, for regression models, were tested with Spearman's rho because variables could not be assumed to be normally distributed.

## Results

Characteristics of the ASD group, the subthreshold ASD group and the non-ASD group in terms of age, gender ratio, number of DSM-5 ASD criteria met, GAF score, IQ and PSI were checked for between-group differences. No differences in gender ratio (*χ*^2^(2, *N* = 90) = 3.11; *P* = 0.21), IQ estimate (*F*(2, 87) = 1.95; *P* = 0.15) or WAIS-IV PSI (*F*(2, 87) = 1.29; *P* = 0.28) were found between the groups. Differences were found between the groups regarding age (*F*(2, 87) = 3.64; *P* = 0.03), number of DSM-5 criteria met (*F*(2, 87) = 162.3; *P* < 0.01) and GAF score (*F*(2, 87) = 4.79; *P* = 0.01). *Post hoc* analysis with Tukey's honestly significant difference test found age to be higher in the subthreshold ASD group compared with the non-ASD group (*P* = 0.03); also, the number of DSM-5 ASD criteria met was higher in the subthreshold ASD group (mean 4.27, s.d. = 0.47) compared with the non-ASD group (mean 1.85, s.d. = 1.13; *P* < 0.01), and higher in the ASD group (mean 5.40, s.d. = 0.69) compared with the subthreshold ASD group (*P* < 0.01). GAF scores were lower in the ASD group compared with the non-ASD group (*P* < 0.01). No differences were found in GAF scores between the ASD group and the subthreshold ASD group (*t*_(61)_ = −0.115, *P* = 0.91).

Based on these results, the ASD group and the subthreshold ASD group were merged into one ‘merged ASD’ group. No differences were found in age (*t*_(88)_ = −1.24, *P* = 0.22), gender ratio (Fisher's exact *P* = 0.48) or IQ estimate (*t*_(88)_ = 1.97, *P* = 0.052) between the merged ASD group and the non-ASD group.

Primary reasons for psychiatric referral are shown in Table 1.

**Table 1:** Primary reasons for psychiatric referrals by autism spectrum disorder classification

Primary referral reason	Merged ASD group (*n* = 63)	Non-ASD group (*n* = 27)
Neurodevelopmental disorder (including ASD)	26 (41%)	10 (37%)
Depression/mood disorder	15 (24%)	6 (22%)
Anxiety disorder	8 (13%)	3 (11%)
Severe or post-traumatic stress	3 (5%)	1 (4%)
Other^a^	4 (6%)	4 (15%)
Not confirmed	7 (11%)	3 (11%)

ASD, autism spectrum disorder.

a. Personality disorder, eating disorder, suspected psychotic disorder, substance or alcohol addiction, exhaustion.

### Sociodemographic characteristics

As shown in Table 2, no differences were found between the merged ASD group and the non-ASD group in terms of any relationship variable, living situation or vocational status.

**Table 2:** Self-reported sociodemographic characteristics

	Merged ASD group (*n* = 63)	Non-ASD group (*n* = 27)	*P*-value
	*n* (%)	*n* (%)	
Employed	39 (62%)	16 (59%)	0.8 (Fisher's exact test)
Highest academic level achieved			0.5 (*χ*^2^-test)
University degree	5 (8%)	4 (15%)	
High school/upper secondary school	39 (62%)	18 (66%)	
Primary school/compulsory school	18 (28%)	5 (19%)	
No primary school degree	1 (2%)	0 (0%)	
Current living situation			0.9 (*χ*^2^-test)
Independent living	47 (75%)	19 (70%)	
Living with parent	13 (21%)	6 (22%)	
Other	3 (4%)	2 (8%)	
Relationship variables			
Married or living with partner	21 (35%)	11 (41%)	0.6 (Fisher's exact test)
Ever been in a romantic relationship^a^	50 (79%)	22 (81%)	1.0 (Fisher's exact test)
Has close friends^b^	43 (68%)	20 (74%)	0.1 (Fisher's exact test)
Support/services			
Extra support in school	26 (41%)	12 (44%)	0.5 (Fisher's exact test)
Current support from social services	7 (11%)	1 (4%)	0.4 (Fisher's exact test)
Past support from social services	22 (35%)	4 (15%)	0.08 (Fisher's exact test)
Ever been placed in special residential home for young people	7 (11%)	1 (4%)	0.4 (Fisher's exact test)
Psychiatric history			
Earlier psychiatry contact	37 (59%)	15 (56%)	0.8 (Fisher's exact test)
Earlier compulsory care	3 (5%)	1 (4%)	1.0 (Fisher's exact test)
Earlier psychiatric in-patient care	11 (18%)	4 (15%)	1.0 (Fisher's exact test)

Bonferroni-corrected *α*-level (0.05/12) = 0.004. ASD, autism spectrum disorder.

a. Self-reported ‘Have you ever been in a long-term romantic relationship (more than 3 months)?’. This includes current marriage or current partner.

b. Self-reported ‘Persons that you spend time with on a regular basis and feel that you can trust’.

### ASD symptoms and parent-rated developmental history

ASDI scores were higher in the merged ASD group (mean 23.5, s.d. = 6.0) than the non-ASD group (mean 7.4, s.d. = 4.9) (*t*_(88)_ = −12.27, *P* < 0.01). For distribution and between-group comparisons of the DSM-5 and Gillberg & Gillberg's criteria for Asperger syndrome, see Table 3. Clear differences were found in self-reported lifelong symptoms of ASD.

**Table 3:** DSM-5 autism spectrum disorder criteria and Gillberg & Gillberg's criteria for Asperger syndrome met in the two groups

	Merged ASD group (*n* = 63) *n* (%)	Non-ASD group (*n* = 27) *n* (%)	Fisher's exact *P*-value
DSM-5 criteria for ASD			
A1, social reciprocity	60 (95%)	7 (26%)	<0.001
A2, non-verbal communication	60 (95%)	9 (33%)	<0.001
A3, relationship deficits	57 (90%)	8 (30%)	<0.001
B1, stereotypical behaviour	1 (2%)	0 (0%)	1.00
B2, rigidity	49 (78%)	6 (22%)	<0.001
B3, limited interests	50 (79%)	4 (15%)	<0.001
B4, sensory reactivity	54 (86%)	16 (59%)	0.004
Gillberg & Gillberg's criteria for Asperger syndrome			
Lifelong severe impairments in reciprocal social interaction	57 (90%)	5 (19%)	<0.001
Lifelong all-absorbing interest pattern	60 (95%)	7 (26%)	<0.001
Lifelong imposition of routines, rituals and interests	59 (94%)	7 (26%)	<0.001
Speech and language peculiarities	44 (70%)	1 (4%)	<0.001
Non-verbal communication problems	63 (100%)	18 (67%)	<0.001
Lifelong motor clumsiness	33 (52%)	8 (30%)	0.07

Bonferroni-corrected *α*-level (0.05/15) = 0.003. ASD, autism spectrum disorder.

Fifty-seven of the 90 total participants (63%) agreed to receive the FTF form and forward it to their parents, and 31 (34% of all 90 participants and 49% of those that received the FTF) of the FTF forms were completed and returned. Twenty-four participants in the merged ASD group and seven in the non-ASD group provided a completed FTF. No differences were found between participants who returned and did not return a competed FTF in terms of age (*t*_(88)_ = −1.31, *P* = 0.19), GAF score (*t*_(88)_ = −0.00, *P* = 0.99) and ASDI result (*t*_(88)_ = 0.81, *P* = 0.42).

On the FTF, the merged ASD group (*n* = 24) had a mean of 4.0 (s.d. = 3.0) domains above the 90th percentile, whereas the non-ASD group (*n* = 7) had a mean of 3.6 (s.d. = 2.4). Mean scores from the 24 proposed items of the FTF-Brief^50^ were 12.6 (s.d. = 10.0) for the merged ASD group and 10.0 (s.d. = 7.2) for the non-ASD group. No between-group comparisons were made because of the low number of respondents in the non-ASD group.

### Psychiatric disorders

Differences between the two groups regarding current psychopathology (across DSM-5 categories) were found regarding non-ASD neurodevelopmental disorders (ADHD and/or any tic disorder) and anxiety disorders (see Table 4). Occurrence of ADHD of any subtype was found in 42 participants (67%) in the merged ASD group and 11 participants (41%) in the non-ASD group. No between-group differences were found when ‘bipolar and related disorders’ and ‘depressive disorders’ were collapsed into a common super-category of ‘mood disorder’ (Fisher's exact *P* = 0.058).

**Table 4:** Current psychiatric disorders at the time of the interview, grouped by DSM-5 categories, presented by autism spectrum disorder classification with between-group comparisons

	Merged ASD group (*n* = 63) *n* (%)	Non-ASD group (*n* = 27) *n* (%)	Fisher's exact *P*-value
Non-ASD neurodevelopmental disorders^a^	57 (90%)	14 (52%)	<0.001
Schizophrenia spectrum and other psychotic disorders	3 (5%)	1 (4%)	1.00
Bipolar and related disorders	18 (29%)	7 (26%)	1.00
Depressive disorders	25 (40%)	5 (19%)	0.056
Anxiety disorders^a^	55 (87%)	10 (37%)	<0.001
Obsessive–compulsive and related disorders	33 (52%)	8 (30%)	0.065
Trauma- and stressor-related disorders	13 (21%)	1 (4%)	0.056
Feeding and eating disorders	6 (10%)	3 (11%)	1.00
Possible gender dysphoria^b^	3 (5%)	0 (0%)	0.558
Disruptive, impulse-control and conduct disorders	7 (11%)	2 (7%)	0.719
Hazardous or harmful alcohol use or drug-related problems	19 (30%)	8 (30%)	1.00

ASD, autism spectrum disorder.

a. Significant at Bonferroni-corrected *α*-level (0.05/11) = 0.0045.

b. Meeting gender dysphoria criterion A1 (‘A marked incongruence between one's experienced/expressed gender and primary and/or secondary sex characteristics’).

#### Co-occurring psychiatric disorders

The merged ASD group had significantly higher numbers of co-occurring non-mood disorders (mean 4.1, s.d. = 1.7) than the non-ASD group (mean 2.0, s.d. = 1.9) (*t*_(88)_ = 5.17, 95% CI 1.29–2.91; *P* < 0.01, *d* = 1.19).

### Outcome

GAF scores were lower in the merged ASD group (mean 49.5, s.d. = 6.4) than the non-ASD group (mean 54.9, s.d. = 9.6) (*t*_(88)_ = −2.66, 95% CI −9.46 to −1.27; *P* = 0.01, *d* = −0.73).

#### Outcome predictors in the merged ASD group

Correlations with GAF score were found in occurrence of current mood disorder, occurrence of ADHD (combined presentation) and number of current non-mood disorders (excluding ADHD) (see Table 5).

**Table 5:** Correlations of possible associated factors with Global Assessment of Functioning score in the merged autism spectrum disorder group (*n* = 63)

	Spearman's rho	*P-*value
Current mood disorder^a^	−0.50	<0.001
Number of current non-mood disorders (excluding ADHD)^a^	−0.43	<0.001
Age	0.20	0.88
Gender	0.07	0.96
WAIS-IV processing speed	0.25	0.05
IQ estimate	0.24	0.05
ADHD (combined presentation)^a^	−0.35	0.004
ADHD (inattentive presentation)	−0.08	0.52
ADHD (hyperactive presentation)	−0.13	0.32
ASDI score	−0.22	0.08

ADHD, attention-deficit hyperactivity disorder; WAIS-IV, Wechsler Adult Intelligence Scale, Fourth Edition; ASDI, Asperger Syndrome (and high-functioning autism) Diagnostic Interview.

a. Significant correlation at Bonferroni-corrected α-level (0.05/10) = 0.005;

Based on the significant correlations, a regression model was created using stepwise selection of variables. The best possible regression model (*F*(2, 60) = 12.29, *P* < 0.01) consisted of current mood disorder (*B* = −5.07, 95% CI −8.07 to −2.07) and number of current non-mood disorders, excluding ADHD (*B* = −0.95, 95% CI −1.87 to −0.03), and explained 26.7% of the variance in GAF score in the merged ASD group.

## Discussion

In this study, we performed in-depth examinations of autistic adult psychiatric out-patients in terms of co-occurring non-ASD neurodevelopmental disorders, other psychiatric disorders, sociodemographic characteristics, alcohol/drug consumption, functional level, ASD symptoms and retrospective parent-rated developmental history. We compared our results with those found in patients without ASD. The participants with ASD met the full DSM-5 criteria for ASD, and a smaller group of participants met the criteria for subthreshold ASD (defined as meeting two out of three necessary A-criteria and at least two B-criteria of ASD according to the DSM-5). Because of the overlap in several features, the ASD group and the subthreshold ASD group were combined into the merged ASD group.

The symptom descriptions from the participants themselves, from parent reports (when available) and from clinical assessments (performed by clinicians experienced in ASD diagnosis) showed that the merged ASD group had lifelong deficits in social-emotional reciprocity, non-verbal communication behaviours and developing, maintaining and understanding relationships. Furthermore, difficulties related to insistence on sameness, inflexible adherence to routines or ritualised patterns of behaviours, highly restricted interests abnormal in intensity or focus, and hyper-/hypo-reactivity to sensory input were also evident. All participants in the merged ASD group reported lifelong experience of their symptoms of ASD. We want to focus on five major findings from the current study.

First, no differences were found in any of the explored sociodemographic characteristics between the merged ASD group and the non-ASD group. This may possibly suggest that social, economic and educational backgrounds in autistic adult psychiatric patients do not differ from non-autistic adult psychiatric patients. This finding is surprising, particularly regarding social relationships, which typically are more limited among autistic persons. As the data is self-reported, it is possible that differences in the definition of relationships can mask possible true differences. Autistic persons might define ‘close friend’ or ‘romantic relationship’ differently from non-autistic persons.^55,56^ On the other hand, there is a well-documented co-occurrence of psychiatric morbidity and limited social relations.

Second, the data from the parental ratings of developmental history (the FTF questionnaire) could suggest that neurodevelopmental disorders are common in adult psychiatry in general. This could not be verified using statistical analyses because of the low number of respondents, but is supported by recent research in adult out-patient psychiatry.^10^ A high prevalence of neurodevelopmental disorders has also been found in studies of other psychiatric populations; significant overrepresentation of neurodevelopmental disorders occur in child and adolescent mental health services,^57^ forensic psychiatry^58^ and criminal justice services.^59^

Third, participants in the merged ASD group had more neurodevelopmental disorders and anxiety disorders compared with the non-ASD group. This was expected, as it has been previously reported that autistic persons have high rates of co-occurring neurodevelopmental disorders.^24^ The higher occurrence of anxiety disorders has been previously reported,^27^ and anxiety disorders (as well as mood disorders) are reported to be the most common co-occurring psychiatric conditions to ASD in adulthood.^26,27,30^ The participants in this study were all adult psychiatric patients within the normal intelligence range, and only a tiny fraction of the ASD group had had a clinical diagnosis of ASD before the study. As previously stated, the research on contributing factors for receiving a diagnosis of ASD at a later age is still inconclusive,^14^ although many of the factors listed in different studies are factors concurrent with our sample of autistic adult psychiatric out-patients, such as less clear ASD symptoms, female sex and occurrence of additional diagnoses. Most participants in this study did not report extra support in school, suggesting that they were not recognised by school staff as having significant difficulties. It might be possible that autistic individuals with less clear symptoms (perhaps with the ability to ‘copy’ prosocial behaviours) have enough social perception to understand that something is different in their social functioning (‘I just never could handle social situations and I have no idea why’, stated in different variants by most of the study participants in the merged ASD group). Experiences of repeated social failures and of feeling different and unable to connect with peers could perhaps be risk factors for developing psychiatric symptoms or disorders.

Fourth, in addition to the greater occurrence of anxiety disorders, the merged ASD group also had significantly more co-occurring psychiatric diagnoses compared with the non-ASD group. This is supported by previous research,^27^ and might imply that the clinical psychiatric picture is more muddled in autistic persons. This would mirror the authors’ clinical experience.

Fifth, GAF score was lower in the merged ASD group compared with the non-ASD group, which also is consistent with previous research. The number of co-occurring psychiatric non-mood diagnoses and current/ongoing mood disorder explained 26.7% of the variability in GAF scores in the merged ASD group, which may indicate that greater disability is mostly caused by the co-occurring psychiatric disorders. Although this is consistent with the concept of ‘autism plus versus autism pure’,^24^ the overlap of ASD, depression and anxiety (87% of our merged ASD group) may index a distinct category or subgroup within the ASD population as a whole.

The results suggest that ASD should be ruled out as an ‘underlying’ disorder in adult psychiatric patients, possibly by collecting a developmental history. Furthermore, there is a need for careful clinical assessment of co-occurring psychiatric disorders in autistic adults within adult psychiatric care services. It is possible that treatment of comorbid psychiatric disorders is the most important way of increasing the functional level in autistic adult psychiatric out-patients. The similarities between autistic and non-autistic adult psychiatric out-patients seem to outweigh the differences, although clear differences exist in clinically assessed symptoms of ASD. This calls for a raised awareness of ASD and increased competency regarding ASD in adult psychiatric services.

### Strengths and limitations

Limitations of this study include the small sample in the comparison group (the non-ASD group). The low participation rate has implications for the representativity of the sample, and limits generalisability of the findings. The lack of parent-reported developmental history in a significant proportion of the study group could have led to an over- or underassessment of ASD in the sample. Although it is possible that the final clinical sample might be an over-selection of ASD cases,^13^ we believe that the examined groups are likely to be typical of non-psychotic patients in non-geriatric adult out-patient psychiatric services. The main strength of the study is the in-depth clinical assessments using validated instruments, performed by experienced clinicians.

## Data Availability

The data that support the findings of this study are available from the corresponding author, J.N., upon reasonable request. The data are not publicly available due to their containing information that could compromise the privacy of research participants.
